# Paralysis of the Muscles of Deglutition

**Published:** 1852-01

**Authors:** Alfred C. Post

**Affiliations:** Professor of Surgery in the University Medical College


### Part 3—Selections.

ARTICLE I.

*Paralysis of the Muscles of Deglutition.* By Alfred C. Post, M. D., Professor of Surgery in the University Medical College.

On the 28th July, 1851, I was called to see Mr. M., a strong, muscular man, from whom I received the following history of his case. The patient was an omnibus driver, 35 years of age, accustomed to the free, but not intemperate use of alcoholic drinks. Five days before I saw him, the weather being quite warm, he attempted to drink a glass of ginger pop, but found himself utterly unable to swallow, the fluid being rejected, partly through the mouth and partly through the nose. The attack was sudden, without any premonition. He was conscious at the same time of a little confusion of mind, of slight thickness of speech, and of a slight degree of numbness in the left side of his body. He sent for a physician, who directed leeches to be applied to his temples, and a blister to his throat, and ordered nutritious enemata of broth. When I saw him, there was continued inability to swallow, the effort being followed by a prolonged fit of coughing, and the ejection of a considerable quantity of extremely viscid mucus. There was no distortion of the countenance, no loss of power in the limbs, but a very slight sense of numbness in the left arm and leg. There was a little thickness of speech, more marked at some times than at others. There was no positive headache, but an uncomfortable feeling about the head, not referred to any particular part. The right pupil was moderately dilated, and the left contracted. The patient and his wife stated that this disparity in the size of the pupils had always existed. The face was always flushed; temperature of the surface natural; pulse abont 100, rather small, and easily compressible; urinary secretion and excretion normal; bowels constipated. I directed the patient to be cupped upon the temples, and to have a current of electricity, to be passed from the back of the neck to the larynx.

29th. Same condition. Repeat electricity. The hair having been shaved on the top of the head, I applied nitric acid along the course of the sagittal suture, extending the application forwards over the Os Frontis, and backwards as far as the protuberance of the Os Occipitis.

30th. No change in the condition of the patient. Ordered an ointment, composed of three grains of strychnine rubbed up with an ounce of cerate; a portion as large as a pea to be rubbed on each side of the neck morning and evening.

Aug. 2d. The patient remaining in the same condition, and having for ten days been sustained exclusively by nutritious enemata, I introduced a catheter into the æsophagus, and through it I gradually injected into the stomach about a pint of beef soup, which produced the comfortable sensation usually followihg the indigestion of food.

About the middle of the night succeeding this day, he lost his consciousness, and at the same time began to perform the most violent and frantic muscular movements, and bellowed so lound that the sound could be heard at a considerable distance from the house. I was called to him in the night, and saw him about an hour after the commencement of the attack. He remained entirely unconscious, struggling with great violence to throw his limbs in different directions, and screaming and yelling in a most frightful manner, but without uttering any articulate sound. His muscular movements were performed with such violence as to require three or four men to keep him from injuring himself. I sent for sulphuric ether, and, having moistened a towel with it, held it over his mouth and nostrils. He struggled against it for a number of minutes, and then gradually became passive. After about six ounces of ether had evaporated, his breathing became stertorous, and the inhalation of the vapor discontinued. He soon fell into a quiet sleep, a free perspiration broke out upon the surface, and the respiration was easy and natural. At 3 A. M., I lay down in an adjoining room, and slept until between 7 and 8 in the morning. I found the patient sitting up on the floor, talking with his wife. His speech was a little more confused than it had been ; his conversation, although calm, was slightly irrational. I directed blisters to be applied to the calves of his legs. In the evening, I saw him again, and found him more composed, lying in bed. In the course of the morning, he had swallowed three tumblers of water, almost without any dfficulty; in the afternoon, his deglutition had again become difficult, although by an effort, he could succeed in swallowing a little fluid.

4th. His blisters were not drawn well ; directed fresh ones to be applied. He is weaker than last evening, and his manner somewhat more composed.

5th. 10 A. M. Patient has been restless through the night, he is quite delirious this morning. He raises himself upon his elbow with difficulty, and holds a chord attached to his bed-post, thinking that he is driving horses ; he also picks at the bed-clothes. His manner is hurried and agitated, and his language incoherent. His muscular strength is much prostrated. He died at about 1 P. M.

I regret that permission could not be obtained to make a post-mortem examination. The case of the autopsy is necessarily imperfect, but even with this defect, it is interesting, as an example of complete paralysis, evident of cerebral origin, almost entirely limited to the muscles of deglutition.—*N. York Med. Times.*

